# Relationships between pain, physical activity and sleep quality among older adults with radiographic knee osteoarthritis: findings from the Hertfordshire Cohort Study

**DOI:** 10.1007/s40520-025-03238-7

**Published:** 2025-11-25

**Authors:** Fiona Kirkham-Wilson, Leo Westbury, Gregorio Bevilacqua, Faidra Laskou, Nicholas Fuggle, Elaine Dennison

**Affiliations:** 1 MRC Lifecourse Epidemiology Centre, University of Southampton, Southampton, UK; 2 NIHR Southampton Biomedical Research Centre, University of Southampton and University Hospital Southampton NHS Foundation Trust, Southampton, UK; 3 Victoria University of Wellington, Wellington, New Zealand

**Keywords:** Knee osteoarthritis, Poor sleep quality, Physical activity, Knee pain, Older adults

## Abstract

**Aims:**

To determine if the relationship between joint pain and sleep quality among individuals with osteoarthritis (OA) differs according to physical activity level among older adults.

**Methods:**

169 community-dwelling older adults in the UK Hertfordshire Cohort Study (aged 71–80) with radiographic knee OA completed a questionnaire. This included: the Western Ontario and McMaster Universities Osteoarthritis Index (WOMAC); the Pittsburgh Sleep Quality Index (PSQI); the Longitudinal Aging Study Amsterdam Physical Activity Questionnaire; and the Hospital Anxiety and Depression (HAD) Scale. Logistic regression was used to examine the WOMAC knee pain score in relation to having poor sleep quality (PSQI > 5) with adjustment for sex, age, and anxiety and depression scores; analyses were performed with and without stratification by physical activity category (bottom sex-specific tertile vs. not).

**Results:**

Knee pain prevalence (WOMAC pain score > 0) was 40.7% among men and 46.6% among women; 37.0% of men and 50.0% of women reported poor sleep quality (PSQI > 5). Higher WOMAC pain scores were related to increased risk of poor sleep quality; odds ratio (95% CI) per unit increase in pain score: 1.15 (1.01,1.32), *p* = 0.038). Relationships were similar across physical activity levels.

**Conclusions:**

Relationships between joint pain and poor sleep quality among older adults with radiographic knee OA were similar, regardless of physical activity level. Our results highlight the high prevalence of both sleep disturbance and significant knee pain in this group, illustrating the need to consider supportive measures as appropriate in this population.

## Background

Osteoarthritis (OA) is the most common joint condition globally, with incidence increasing with age [1]. Historically OA was considered a condition of ‘wear and tear’, but its pathogenesis is now recognized to be complex and multi-factorial, involving progressive dysregulation of the structures within the joint [2]. Szebenyi et al. undertook a cohort study demonstrating that radiographic knee OA does not necessarily cause pain, but the more compartments of the knee with OA, the higher the risk of having pain [3]. Severe radiographic changes at the knee affect nearly 50% in those 75 years and above. Among participants aged over 45 years in the Framingham Study, the prevalence of radiographic knee OA was 19.2% and, in those over 80 years, the figure rose to 43.7% [4]. While pain limiting sleep is recognized as an indication for referral for joint replacement, this would typically occur with advanced radiographic change.

Sleep patterns change with ageing; difficulties falling asleep, staying asleep, and waking tired are among the main complaints [5]. The implications of poor sleep for health are well documented, and older adults are already at increased risk of poorer function (both mental and physical) associated with ageing [6]. Poor sleep is common in those with OA [7], and sleep typically worsens with increasing severity of OA [8]. A polysomnographic study by Leigh et al. identified that people with OA had more stage 1 sleep (light sleep) compared to matched controls without OA who benefitted from deeper sleep [9], suggesting that OA impacts not only the amount of sleep but also the quality. The reason for sleep disturbance in OA is unclear, but pain is likely to be a strong contributory factor.

Anecdotally however higher physical activity (PA) levels are associated with better sleep [10]. Chien & Chen found that poor sleep quality was independently associated with physical disability, after adjusting for co-morbidities [11]. Song et al. considered relationships between PA, sleep and OA in adults aged 45–79 years at baseline, finding lower levels of PA at baseline were associated with worse sleep and more severe OA by the end of an eight-year follow-up [12].

However, little is known regarding whether the relationship between joint pain severity and sleep quality among individuals with OA differs according to physical activity levels. Therefore, we investigated this in the Hertfordshire Cohort Study (HCS), a population-based cohort of community-dwelling older people. Demonstration of better sleep among those individuals with knee OA who remained physically active may provide motivation to patients who may otherwise struggle to remain active.

## Methods

### The Hertfordshire Cohort Study

HCS is a population-based study of men and women who were born in Hertfordshire (UK) from 1931 to 1939. The original aim of this cohort study was to examine the relationship between growth in infancy and risk of chronic diseases in adulthood. Potential participants were first identified through historical birth ledgers at the Hertfordshire county office. These ledgers contained information on birth weight, illnesses, development, and infant feeding from birth to one year of age. Exclusion criteria were multiple births, childhood deaths, records with missing birth weight or weight at one year, or individuals with insufficient tracing information. HCS comprises 1579 men and 1418 women who were still alive and registered with a Hertfordshire General Practitioner in 1998. From 1998 to 2004, HCS participants completed a home interview and attended a research clinic for a comprehensive assessment of their sociodemographic, lifestyle, and clinical characteristics [13, 14].

At baseline (1998–2004), 966 participants from East Hertfordshire underwent musculoskeletal phenotyping. In 2011, 443 agreed to participate in a follow-up study. The analysis sample for this article consists of 169 participants who had radiographic knee osteoarthritis at that time and had data available on sleep quality and physical activity.

### Ascertainment of participant information in 2011

Information was ascertained at the home interview through clinician-administered questionnaires: smoking status, alcohol consumption and average daily outdoor physical activity (Longitudinal Aging Study Amsterdam Physical Activity Questionnaire (LAPAQ) [15]) were ascertained. Participants with physical activity levels in the bottom third of the sex-specific distribution were classified as having low physical activity. The WOMAC, with subscales measuring pain, stiffness, and physical function [8], was also administered; the pain subscale was used in the current analysis. Sleep quality was evaluated using the Pittsburgh Sleep Quality Index (PSQI), with a PSQI score greater than 5 indicating poor sleep quality [16]. Anxiety and depression were assessed using the Hospital Anxiety and Depression Scale (HADS), with separate scores for anxiety and depression [17].

Anterior–posterior and lateral patellofemoral knee radiographs were taken of both knees after the 2011 home visit and joints were graded based on the Kellgren and Lawrence (K&L) grading system [18]. Each participant’s K&L score was taken as the maximum score at either the tibiofemoral or patellofemoral location on either knee. Participants with a K&L score ≥ 2 were classified as having radiographic knee osteoarthritis.

### Statistical methods

Participant characteristics were described using summary statistics. Logistic regression was used to examine WOMAC knee pain score in relation to having poor sleep quality (PSQI > 5) with adjustment for sex, age, and anxiety and depression scores; analyses were performed among the whole sample, and with stratification by physical activity category (bottom sex-specific tertile versus not) and maximum K&L score (maximum score at the tibiofemoral or patellofemoral location on either knee of 2 versus > 2). Sex-interactions regarding the relationship between WOMAC pain score and poor sleep quality were not statistically significant (*p* > 0.05) so men and women were pooled to increase statistical power. Analyses were conducted using Stata, release 15 (StataCorp, College Station, TX, USA); *p* < 0.05 was regarded as statistically significant.

## Results

### Participant characteristics

Participant characteristics of the analysis sample are presented in Table 1. Mean (SD) age was 75.7 (2.6) among men and 75.8 (2.7) among women. Overall, 30 (37.0%) men and 44 (50.0%) women had poor sleep quality (Pittsburgh Sleep Quality Index score > 5). Median (lower quartile, upper quartile) WOMAC pain scores were 0 (0, 2) among men and 0 (0, 4) among women; 33 (40.7%) men and 41 (46.6%) women had knee pain (WOMAC knee pain scores > 0).

**Table 1:** Descriptive statistics of the 169 participants with radiographic knee osteoarthritis who were included in the analysis sample

Characteristic	Mean (SD); median (lower quartile, upper quartile); or *n*(%)	
	Men (*n* = 81)	Women (*n* = 88)
Age (years)	75.7 (2.6)	75.8 (2.7)
Smoking status		
Never	35 (43.2%)	51 (58.0%)
Ex	44 (54.3%)	35 (39.8%)
Current	2 (2.5%)	2 (2.3%)
Alcohol consumption (units per week)	6 (2, 11)	0 (0, 2)
Physical activity in last 2 weeks (min/day) [LAPAQ]	181 (105, 249)	187 (100, 240)
Pittsburgh Sleep Quality Index score	4 (2, 7)	6 (4, 10)
Poor sleep quality (Pittsburgh Sleep Quality Index score > 5)	30 (37.0%)	44 (50.0%)
Anxiety score (HAD)	3 (2, 5)	5 (2, 7)
Depression score (HAD)	3 (1, 5)	3 (2, 4)
WOMAC knee pain score	0 (0, 2)	0 (0, 4)
Pain (WOMAC pain score > 0)	33 (40.7%)	41 (46.6%)

LAPAQ: Longitudinal Aging Study Amsterdam Physical Activity Questionnaire

HAD: Hospital Anxiety and Depression Scale

WOMAC: Western Ontario and McMaster Universities Osteoarthritis Index

### Associations between WOMAC pain scores and sleep quality

Odds ratios for having poor sleep quality (Pittsburgh Sleep Quality Index score > 5) per unit increase in WOMAC knee pain score are shown in Table 2, with and without stratification by physical activity level and maximum K&L score. Higher WOMAC knee pain scores were related to greater likelihood of poor sleep quality. For example, the odds ratio (95% CI) for having poor sleep quality per unit increase in WOMAC knee pain score in the entire sample was 1.15 (1.01,1.32), *p* = 0.038.

**Table 2:** Odds ratios for having poor sleep quality (Pittsburgh sleep quality index score > 5) per unit increase in WOMAC knee pain score among participants with radiographic knee osteoarthritis (stratified according to physical activity level and K&L score)

	Kellgren and Lawrence (K&L) subgroup					
Physical activity subgroup	No K&L stratification		K&L score = 2		K&L score > 2	
	Odds ratio (95% CI)	*P*-value	Odds ratio (95% CI)	*P*-value	Odds ratio (95% CI)	*P*-value
No physical activity stratification	1.15 (1.01,1.32)	0.038	1.16 (1.00,1.34)	0.050	1.10 (0.78,1.56)	0.579
Low physical activity	1.18 (0.96,1.44)	0.116				
Normal physical activity	1.16 (0.96,1.40)	0.130				

Odds ratios were estimated using logistic regression models; all models included sex, age, HAD anxiety score and HAD depression score as adjustments

Low physical activity: bottom third of the sex-specific distribution of the Longitudinal Aging Study Amsterdam Physical Activity score; participants not in this category were regarded as having a normal physical activity level

WOMAC: Western Ontario and McMaster Universities Osteoarthritis Index

Estimates of odds ratios for this association were similar when analyses were stratified according to physical activity level (Table 2). For example, the odds ratio for having poor sleep quality per unit increase in WOMAC knee pain score was 1.18 (0.96,1.44) for individuals with physical activity levels in the bottom sex-specific third of the distribution, and 1.16 (0.96,1.40) for those who had higher physical activity levels. Furthermore, the odds ratio for having poor sleep quality per unit increase in WOMAC knee pain score in the entire analysis sample (1.15 (1.01,1.32)) was similar when restricted to participants with a maximum K&L score of two (1.16 (1.00,1.34)) as shown in Table 2.

## Discussion

In this sample of community-dwelling older people with radiographic knee osteoarthritis, higher WOMAC knee pain scores were related to increased risk of poor sleep quality, with similar relationships observed when stratified according to physical activity level and severity of radiographic knee osteoarthritis. While the study population was relatively inactive, there was no evidence that the association between knee pain and poor sleep quality was weaker among participants with higher physical activity levels. These data highlight the need to address pain management carefully among individuals with knee OA as otherwise a vicious cycle of pain, disrupted sleep, and poor physical function may emerge.

Activity levels in this population were relatively low. Poor sleep quality is itself a risk factor for reduced physical activity; a 2021 cohort study found that participants with 3 or more nights of restless sleep per week were almost 25% less active in terms of moderate physical activity than those who had one or less nights of restless sleep per week [19]. An observational prospective study from 2000 suggested that type of sleep disturbance in OA varied significantly between individuals but the most common was difficulty with sleep maintenance [20]. Poor sleep also worsens the symptoms of pain, including pain remote to the joint(s) affected by OA, suggesting a component of central nervous system augmentation of pain [12]. Further work to establish the mechanisms of these associations may provide treatment targets.

Our study has several limitations. First, a healthy participant effect is evident in the HCS cohort [13], and sample attrition across different follow-up waves may have introduced additional selection effects. This, along with the fact that participants were community-dwelling, may limit the generalisability of findings to other groups of individuals. Nevertheless, the baseline cohort was comparable to participants in the nationally representative Health Survey for England [13]. Second, the sample size was small (*n* = 169) which reduces the statistical power to detect associations, especially in the stratified analyses. In light of this, we focused on effect sizes, rather than p-values, in the reporting of our findings. However, despite the small sample size, our results are biologically plausible and consistent with previous studies [5, 7]. Because of the small numbers of individuals in each level of K&L grading, we cannot be certain that relationships are the same across all OA radiographic gradings. However, relationships were similar when restricted to participants with a K&L grade of 2. Third, WOMAC scores were only available for each individual, rather than for each knee; we used the maximum K&L score from the tibiofemoral or patellofemoral site of either knee in our analysis, which may have led us to understate the strength of associations. Fourth, the cross-sectional design of this study limits the ability to infer causality between knee pain and sleep quality. In addition, potential causal effects of sleep quality on knee pain cannot be excluded. Lastly, physical activity data were self-reported rather than objectively measured, and only information on overall physical activity (from time spent walking, cycling, gardening, playing sport, and housework) was available and not duration of activity at various intensity levels. However, this study benefits from the HCS’s rigorous phenotyping protocols, conducted by well-trained fieldworkers and management by a skilled multidisciplinary team. Additionally, we were able to adjust for participants’ anxiety and depression levels in our analysis.

In conclusion, greater knee pain was related to increased likelihood of poor sleep quality in this study of community-dwelling older people with radiographic knee OA. However, this relationship did not vary according to physical activity level, which was generally low in this study. The high prevalence of poor sleep quality in this population highlights the need to ask about sleep when clinicians interact with OA participants. Further work in larger, more physically active populations is now required.

## Data Availability

The data used in this article cannot be shared widely due to consent restrictions. The participants only consented for their data to be shared with the Hertfordshire Cohort Study Research Team. Requests to access Hertfordshire Cohort Study data for new research projects should be made to ED (emd@mrc.soton.ac.uk).
